# Tuberculosis Acquired Outside of Households, Rural Vietnam

**DOI:** 10.3201/eid1609.100281

**Published:** 2010-09

**Authors:** Tran N. Buu, Dick van Soolingen, Mai N.T. Huyen, Nguyen N.T. Lan, Hoang T. Quy, Edine W. Tiemersma, Martien W. Borgdorff, Frank G.J. Cobelens

**Affiliations:** Author affiliations: Pham Ngoc Thach Tuberculosis and Lung Disease Hospital, Ho Chi Minh City, Vietnam (T.N. Buu, M.N.T. Huyen, N.N.T. Lan, H.T. Quy);; National Institute of Public Health and the Environment, Bilthoven, the Netherlands (D. van Soolingen);; KNCV Tuberculosis Foundation, The Hague, the Netherlands (E.W. Tiemersma);; Academic Medical Centre, Amsterdam, the Netherlands (M.W. Borgdorff, F.G.J. Cobelens)

**Keywords:** Tuberculosis and other mycobacteria, pulmonary, households, transmission, Vietnam, dispatch

## Abstract

Using population-based data from rural Vietnam, we assessed tuberculosis (TB) transmission within and outside of households. Eighty-three percent of persons with recent household TB were infected by different strains of *Mycobacterium tuberculosis* than were their household members. This result argues against the effectiveness of active TB case finding among household members.

Because of airborne transmission of *Mycobacterium tuberculosis*, persons who share a household with persons who have tuberculosis (TB) are at high risk for infection (*1*). In urban settings in South Africa, studies using DNA fingerprinting that found high TB transmission and HIV prevalence up to 5% suggested that more TB transmission occurs outside households than previously assumed (*2**,**3*). Few data exist for other settings that have a high incidence of TB, particularly rural areas in Asia where HIV prevalence is low.

In Vietnam, TB incidence is high; 70% of the population live in rural areas where the average HIV prevalence in adults is <0.5% (*4*). A recent survey showed TB prevalence to be higher than assumed, which suggests that case finding is inadequate (*5*). Improving TB case finding is thus a priority for TB control in Vietnam. To assess the value of active case finding among household contacts of patients with infectious TB, we studied within- and outside-household TB transmission, using data collected in a population-based study in rural southern Vietnam. The characteristics of the study site have been described elsewhere (*6*).

## The Study

We prospectively collected data on all patients who had TB sputum smear–positive samples and for whom TB had been diagnosed during January 1, 2003–December 31, 2006, by the National TB Program by microscopic examination of >1 Ziehl-Neelsen–stained sputum smears (*7*). *M. tuberculosis* isolates were typed by spoligotyping, 15-loci variable number of tandem repeats (VNTR) typing, and partly by IS*6110*-based restriction fragment-length polymorphism (RFLP) typing (*8*). We collected sociodemographic data through structured interviews with individual patients about their households (defined as all persons who share the same floor and the same food).

Index case-patients were defined as all persons for whom TB had been diagnosed through December 31, 2004. From our database, we identified as household case-patients their household members for whom TB was diagnosed within 24 months after enrollment of the index case-patient. We compared the genotypes and DNA fingerprints of strains isolated from household case-patients with strains isolated from index case-patients in the same household. TB was diagnosed passively, i.e., at the time persons sought care at the study clinics because of symptoms.

Isolated strains of *M. tuberculosis* were defined as identical if spoligotype and RFLP and/or VNTR patterns, as applicable, were the same (differing by <1 locus) in the household and index case-patients. Mixed infections were defined as RFLP types with discordant spoligotypes or VNTR patterns with multiple alleles on >1 locus in 1 patient isolate. Genotypes were defined by spoligotyping as described by Brudey et al.; the Beijing genotype was defined as any isolate without direct repeat spacers 1–34 and >3 of the spacers 35–43 (*9*,*10*).

Data were entered into Epi Info version 6.04 (Centers for Disease Control and Prevention, Atlanta, GA, USA). Analyses were performed in Stata version 8 (StataCorp, College Station, TX, USA). Patients with negative cultures or cultures that grew nontuberculous mycobacteria were excluded.

Through December 31, 2004, a total of 1,589 patients were registered. We excluded 36 patients because of mismatching and 20 because of laboratory/technical errors; therefore, sputum specimens of 1,533 patients were cultured. After excluding 57 negative cultures and 34 cultures that grew nontuberculous mycobacteria, we included 1,442 (90.8%) index case-patients in the analyses. These patients had 4,141 household contacts >15 years of age. During 24 months of follow-up, >1 household case-patients were identified for each of 12 (0.8%) index case-patients, including 1 with 2 household case-patients. Household case-patients were not significantly associated with sex, age, family size, *M. tuberculosis* genotype, or smear grade of the index case-patient (data not shown). None of the 12 index and 13 household case-patients had mixed infections.

Of the 13 household case-patients, 1 (a 72-year-old man) had recurrent infection; 3 had infections were caused by Beijing genotype (23%). For 2 (17%) household case-patients (95% confidence interval [CI] 1.9%–45.4%), the infecting strain was similar to that from the index case-patient. The interval between diagnoses for index and household case-patients was (mean ± SD) 13.0 ± 2.8 weeks for identical strains, and 47.1 ± 33.1 weeks for different strains (p = 0.114).

## Conclusions

Of TB case-patients who had had exposure to a TB patient in their household during the preceding 2 years, 17% harbored the same strain as the index case-patient. Even if we classified as similar 1 case pair with only 2 loci differences according to VNTR (no RFLP type available: patient 4 in the Table), i.e., if we assumed that this difference was caused by evolution of the VNTR pattern or a processing error, the proportion of infections acquired outside the household still would be 77% (10/13). Although we cannot exclude the possibility of transmission from another person in the same household for whom TB had not been diagnosed by the TB Program and included in our database, this finding suggests that in our study population, most TB cases resulted from transmission outside the household. These results differ from observations in low-incidence settings but are similar to those in high-incidence settings in South Africa, Gambia, and Malawi (*11**–**14*), even though the follow-up periods of these studies differed. The high proportion of cases resulting from transmission outside the household in those studies and ours may be explained by high exposure to different *M. tuberculosis* strains circulating in the community. Having common factors that determine the risk for breakdown of TB infection to disease also may play a role but is less likely in our study because HIV prevalence is lower in rural Vietnam than in South Africa. Alternatively, the large proportion of nonmatching genotypes within households could reflect specimen mislabeling. Although we excluded specimens that had been mislabeled at collection, additional mislabeling may have occurred in the laboratory. However, because mislabeling is expected to occur at random, for most of the nonmatches to be caused by errors, nearly half of all specimens must have been mislabeled, which seems unlikely.

**Table:** Characteristics of case-patients with exposure to tuberculosis within household, rural Vietnam, 2003–2006*

Patient no.	Age, y/sex	Previous treatment	Relationship to index case-patient	Genotype			Comparison of strain type with that of index case-patient			
				Index case-patient	Household case-patient		Spoligotype (no. different spacers)†	RFLP	VNTR‡	Final classification
1	40/M	No	Brother	Beijing	EAI2-Manila		Different (26)	Different	12	Different
2	55/M	No	Brother	NR	Beijing		Different (27)	Different	10	Different
3	49/M	No	Brother	EAI4-VNM	EAI4-VNM		Same (0)	Different	4	Different
4	51/F	No	Spouse	EAI4-VNM	EAI4-VNM		Same (0)	ND	2	Different
5	48/M	No	Spouse	Zero	Zero		Same (0)	Same	0	Same
6	36/M	No	Brother	Beijing	Beijing		Same (0)	ND	1	Same
7	41/M	No	Grandson	EAI5	EAI5		Same (1)	ND	5	Different
8	41/M	No	Brother	U	EAI4-VNM		Different (17)	ND	2	Different
9	31/M	No	Brother	EAI4-VNM	NR		Different (4)	ND	4	Different
10	29/M	No	Brother	EAI5	NR		Different (5)	ND	7	Different
11	72/M	Yes	Grandson	EAI4-VNM	Beijing		Different (27)	Different	13	Different
12	37/M	No	Son	Beijing	EAI5		Different (29)	ND	7	Different
13	38/F	No	Spouse	EAI4-VNM	EAI2-NTB		Different (16)	Different	3	Different

*RFLP, restriction fragment-length polymorphism; VNTR, variable number of tandem repeats; EAI4-VNM: East African–Indian family, Vietnam genotype; NR, not represented in spoligotype database (*10*); ND, RFLP typing not done. †Difference in the number of direct repeat spacers between the spoligotypes of the index case-patient and that of the household case-patients. Genotypes based on spoligotyping in accordance with classification by Brudey et al. (*10*). ‡No. loci with different alleles.

We identified household TB cases for ≈1% of the index cases, which is less than the 6%–7% reported in studies of active case finding (*1**,**14*). This finding could reflect studies that included household case-patients of all ages with all forms of TB, whereas we included only persons >15 years of age who had smear-positive TB. In addition, we relied on self-reporting rather than on active TB screening. Although self-reporting may have limited the number of household cases we identified, it was unlikely to have affected the proportion resulting from household transmission. An exception should probably be made for young children, who because of less social mixing, may have higher probability of being infected within than outside the household. The shorter interval between diagnoses of index and household case-patients with identical strains, as well as the observation that the average interval for case-patients with different strains was approximately half the 2-year study period (i.e., consistent with random occurrence over time) supports our interpretation that the strains that differed between household and index cases were from another source.

In rural Vietnam, where HIV prevalence is low, most persons with secondary TB are infected by a source case-patient outside of their households. This finding argues against active TB case finding among household members as an effective method for improving case finding.

## References

[1] Wang PD, Lin RS. Tuberculosis transmission in the family. J Infect. 2000;41:249–51. 10.1053/jinf.2000.0736 11120613

[2] Verver S, Warren RM, Munch Z, Richardson M, van der Spuy GD, Borgdorff MW, Proportion of tuberculosis transmission that takes place in households in a high incidence area. Lancet. 2004;363:212–4. 10.1016/S0140-6736(03)15332-9 14738796

[3] Marais BJ, Heseling AC, Schaaf HS, Gie RP, van Helden PD, Warren RM. *Mycobacterium tuberculosis* transmission is not related to household genotype in a setting of high endemicity. J Clin Microbiol. 2009;47:1338–43. 10.1128/JCM.02490-08 19261801 PMC2681880

[4] The Socialist Republic of Vietnam. The third country report on following up the implementation to the declaration of commitment on HIV and AIDS. Hanoi (Vietnam): The Ministry of Health; 2008.

[5] Hoa NB, Sy DN, Nhung NV, Tiemersma EW, Borgdorff MW, Cobelens FGJ. A national survey of tuberculosis prevalence in Vietnam. Bull World Health Organ. 2010;88:273–80. 10.2471/BLT.09.067801 20431791 PMC2855599

[6] Buu TN, Huyen MN, Lan NT, Quy HT, Hen NV, Zignol M, The Beijing genotype is associated with young age and multidrug-resistant tuberculosis in rural Vietnam. Int J Tuberc Lung Dis. 2009;13:900–6. 19555542

[7] World Health Organization. Treatment of tuberculosis. Guidelines for national programmes, 3rd ed. WHO/CDS/TB/2003.313. Geneva: The Organization; 2003.

[8] Kamerbeek J, Schouls L, Kolk A, van Agterveld M, van Soolingen D, Kuijper S, Simultaneous detection and strain differentiation of *Mycobacterium tuberculosis* for diagnosis and epidemiology. J Clin Microbiol. 1997;35:907–14. 9157152 10.1128/jcm.35.4.907-914.1997 PMC229700

[9] Filliol I, Driscoll JR, van Soolingen D, Kreiswirth BN, Kremer K, Valétudie G, Snapshot of moving and expanding clones of *Mycobacterium tuberculosis* and their global distribution assessed by spoligotyping in an international study. J Clin Microbiol. 2003;41:1963–70. 10.1128/JCM.41.5.1963-1970.2003 12734235 PMC154710

[10] Brudey K, Driscoll JR, Rigouts L, Prodinger WM, Gori A, Al-Hajoj SA, *Mycobacterium tuberculosis* complex genetic diversity: mining the fourth international spoligotyping database (SpolDB4) for classification, population genetics and epidemiology. BMC Microbiol. 2006;6:23. 10.1186/1471-2180-6-23 16519816 PMC1468417

[11] Schaaf HS, Michaelis IA, Richardson M, Booysen CN, Gie RP, Warren R, Adult-to-child transmission of tuberculosis: household or community contact? Int J Tuberc Lung Dis. 2003;7:426–31. 12757042

[12] de Jong BC, Hill PC, Aiken A, Awine T, Antonio M, Adetifa IM, Progression to active tuberculosis, but not transmission, varies by *Mycobacterium tuberculosis* lineage in The Gambia. J Infect Dis. 2008;198:1037–43. 10.1086/591504 18702608 PMC2597014

[13] Behr MA, Hopewell PC, Paz EA, Kawamura LM, Schecter GF, Small PM. Predictive value of contact investigation for indentifying recent transmission of *Mycobacterium tuberculosis.* Am J Respir Crit Care Med. 1998;158:465–9. 9700122 10.1164/ajrccm.158.2.9801062

[14] Crampin AC, Glynn JR, Traore H, Yates MD, Mwaungutu L, Mwennebabu M, Tuberculosis transmission attributable to close contacts and HIV status, Malawi. Emerg Infect Dis. 2006;12:729–35. 16704828 10.3201/eid1205.050789 PMC3374426

